# Time Course and Pattern of Metastasis of Cutaneous Melanoma Differ between Men and Women

**DOI:** 10.1371/journal.pone.0032955

**Published:** 2012-03-06

**Authors:** Liljana Mervic

**Affiliations:** 1 Department of Dermatology, Center of Dermatooncology, University of Tüebingen, Germany; 2 Department of Dermatovenereology, University Medical Centre Ljubljana, Slovenia; University of Tennessee, United States of America

## Abstract

**Background:**

This study identified sex differences in progression of cutaneous melanoma.

**Methodology/Principal Findings:**

Of 7,338 patients who were diagnosed as an invasive primary CM without clinically detectable metastases from 1976 to 2008 at the University of Tuebingen in Germany, 1,078 developed subsequent metastases during follow up. The metastatic pathways were defined in these patients and analyzed using the Kaplan-Meier method. Multivariate survival analysis was performed using Cox modeling. In 18.7% of men and 29.2% of women (*P*<0.001) the first metastasis following diagnosis of primary tumor was locoregional as satellite/in-transit metastasis. The majority of men (54.0%) and women (47.6%, *P* = 0.035) exhibited direct regional lymph node metastasis. Direct distant metastasis from the stage of the primary tumor was observed in 27.3% of men and 23.2% of women (*P* = 0.13). Site of first metastasis was the most important prognostic factor of survival after recurrence in multivariate analysis (HR:1.3; 95% CI: 1.0–1.6 for metastasis to the regional lymph nodes vs. satellite/in-transit recurrence, and HR:5.5; 95% CI: 4.2–7.1 for distant metastasis vs. satellite/in-transit recurrence, *P*<0.001). Median time to distant metastasis was 40.5 months (IQR, 58.75) in women and 33 months (IQR, 44.25) in men (*P* = 0.002). Five-year survival after distant recurrence probability was 5.2% (95% CI: 1.4–2.5) for men compared with 15.3% (95% CI: 11.1–19.5; *P* = 0.008) for women.

**Conclusions/Significance:**

Both, the pattern of metastatic spread with more locoregional metastasis in women, and the time course with retracted metastasis in women contributed to the more favorable outcome of women. Furthermore, the total rate of metastasis is increased in men. Interestingly, there is also a much more favorable long term survival of women after development of distant metastasis. It remains a matter of debate and of future research, whether hormonal or immunologic factors may be responsible for these sex differences.

## Introduction

Cutaneous melanoma (CM) is considered to have a high malignant potency. Metastatic spread may arise from very small tumors. At a time when the incidence of many tumor types is decreasing, melanoma incidence continues to increase. Mortality figures over the past decades show trends that differ from those for incidence, with a much lower rate of mortality increase than that recorded for incidence [1]. Nevertheless, at the start of the 21st century, CM remains a potentially fatal malignancy. The most important factor for successful management of melanoma is early diagnosis, allowing treatment to be undertaken at a stage when cure is readily achievable. Malignant melanoma in its advanced stage of metastatic disease has a poor prognosis with a median survival time of approximately 8 months [2].

Significant differences exist between male and female patients in melanoma incidence, presentation of primary tumor, and mortality [3]–[5]. Despite differences in incidence between sexes across continents with a greater incidence among woman in Europe and predominance of men in Australia and United States, the survival advantage for women is consistently observed independently of the incidence pattern [3]–[6]. It has been shown that men have worse primary tumors and experience shorter survival than women [3]–[5]. The differences in tumor thickness, ulceration and body site distribution between sexes can explain the sex differences in survival of melanoma to a certain degree, however, large body of evidence observed sex to remain an independent significant predictor of survival after adjusting for these factors [4]–[8].

Melanoma can metastasize either by the lymphatic or by the hematogenous route. Melanoma metastases develop via three main metastatic pathways, beginning with either satellite or in-transit metastases, up to 2 cm from the primary tumor or in the skin between the site of the primary tumor and the first lymph node, respectively, with regional lymph node metastasis, or with distant metastasis. About half of all patients with tumor progression develop regional lymph node metastasis as first metastasis. In about one third primary development of distant metastasis is observed and in the remaining patients, satellite or in-transit metastases are the site of the first recurrence [9], [10]. Little is currently known of factors that might influence which type of recurrence occurs.

The primary objective of this study was to analyze the time course of melanoma progression and three different metastatic pathways for the spread of CM, divided into satellite or in-transit metastasis, regional lymph node metastasis, and distant metastasis, with a particular emphasis on the differences between sexes. A research of sex-related differences in progression of CM may contribute to our understanding of the observed differences in survival between men and women.

## Methods

From 1976 to 2008, the German Central Malignant Melanoma Registry (CMMR) recorded the data of 9,044 melanoma patients (4,221 men and 4,823 women) treated at the Tübingen University Department of Dermatology in southern Germany. For this study, only the patients from the Center in Tübingen were included, because in this region the registration of patients reaches the completeness of population based registries [11]. All patients had given their written informed consent to have their data on primary tumor and follow-up recorded within the CMMR. Approval for this retrospective analysis was obtained by the Ethics commitee Tübingen, Germany (approval number 655/2011A). Patient data were analyzed anonymously. Approval for this study was gained retrospectively.

The CMMR is a hospital-based registry. Written informed consent was obtained from all patients in this study. The aims and methods of data collection by the CMMR have previously been reported in detail [12], [13]. Data obtained for each patient included sex, date of birth, date of diagnosis, anatomical location and histopathological characteristics of the primary tumor, date of diagnosis and site of recurrence, date of the last follow-up examination, and (if a patient died) the date and cause of death. The patient sample was divided into three different age groups: 43 years or younger, 44–60 years old, and more than 60 years old. The anatomical sites of the primary tumors were divided into four groups: head and neck, trunk, upper extremities, and lower extremities. The tumor thickness was divided into four groups according to the Tumor Node Metastasis classification of the American Joint Committee on Cancer: less than or equal to 1, 1.01–2, 2.01–4, and more than 4 mm. The level of invasion was classified according to the system described by Clark. The following histopathological subtypes were distinguished: superficial spreading melanoma, nodular melanoma, lentigo maligna melanoma, and acral lentiginous melanoma. The site of recurrence was categorized into three groups: satellite or in-transit recurrence, metastasis to the regional lymph nodes, and metastasis to distant anatomical sites. Satellite metastasis is defined as the development of metastatic nodules within 2 cm of the primary tumor. In-transit metastasis develops within the metastatic drainage area before the first regional lymph node basin. Different metastatic pathways in the progression of CM were analyzed. A metastatic pathway was taken to be the first pathway of spread in disease progression. Three pathways were defined: satellite/in-transit metastases, regional lymph node metastasis, and distant metastasis. The decades of diagnosis were classified as follows: 1970–1979, 1980–1989, 1990–1999, and 2000–2008.

All patients underwent a physical examination, blood tests, abdominal ultrasound scans, and chest X-rays at the time of the initial diagnosis and were thereafter regularly followed. The analysis included only patients with an invasive primary CM without clinically detectable metastases after all investigations performed at the time of primary diagnosis who later on developed metastases. Patients with ocular, mucosal, and in-situ melanomas, patients younger than 14, and those with a follow-up of less than 3 months were excluded from the analysis. 7,338 patients (3,347 men and 3,991 women) were diagnosed as an invasive primary CM without clinically detectable metastases (Stages I and II, American Joint Committee on Cancer). Among these patients, 517 women and 561 men developed subsequent metastases during 10-year follow up and were included into the analysis.

### Statistical analysis

A χ^2^ test was used to compare the distribution of categorical variables between groups. The Mann-Whitney test was used to compare continuous variables between groups. The follow-up times were defined. Recurrence-free survival (RFS) time was defined as the date of diagnosis of first recurrence minus the date of diagnosis of primary tumor. Distant recurrence-free survival (DRFS) time was defined as the date of diagnosis of distant metastasis minus the date of diagnosis of primary tumor. Survival-after-recurrence (SAR) time was defined as the date of the last follow-up or death minus date of diagnosis of first recurrence, and survival-after-distant-recurrence (SADR) time as the date of the last follow-up or death minus date of diagnosis of first distant metastasis. Follow-up time was cut to a maximum of 10 years because in this period patients usually participate in the CM follow-up program. Only deaths caused by CM were considered in the analysis of SAR and SADR, and deaths from other causes were regarded as censored events. In the analysis of RFS and DRFS, deaths due to melanoma were regarded as recurrence if no other event had been documented. The patients who died from melanoma were nearly all treated at the Tuebingen University Department of Dermatology and melanoma deaths are very unlikely to be under-reported. Deaths of causes not otherwise specified were regarded not to be caused by melanoma and regarded as censored. The follow-up time was described as a median value with an interquartile range (IQR). All 1,078 patients with an invasive primary CM at the time of diagnosis (clinically and histopathologically staged) who developed metastases during follow up were included in survival analysis. Probability of metastasis in the group comparison and melanoma-specific survival curves and estimated survival rates with relative 95% confidence intervals (CIs) were generated using the Kaplan-Meier method and were compared using a two-sided log-rank test. In the multivariate analysis, age, tumor thickness, SAR time, and the decades of diagnosis were entered as continuous variables. Categorical variables were dummy-coded. The Cox model was described by means of hazard ratios (HRs) together with 95% CIs, and *P* values were based on the Wald test.

Throughout the analyses, two-sided *P* values of less than 0.05 were considered statistically significant. Statistical analyses were carried out using the Statistical Package for the Social Sciences (SPSS) V.15.0.1 (SPSS, Chicago, Illinois, USA).

## Results

### Natural course of melanoma

During follow-up of 7,338 patients who were diagnosed at a stage of an invasive primary CM without clinically detectable metastases, disease progression was noted in 1,078 patients. More men (561, 16.8%) than women (517, 13.0%, *P*<0.001) with primary CM at diagnosis developed metastases. A total of 414 (12.4%) men and 348 (8.7%, *P*<0.001) women in our study group had developed distant metastasis by the time of the analysis. Various clinical and histopathological characteristics of the 1,078 melanoma patients who progressed from the stage of primary invasive CM at the time of diagnosis to the stage of regional or distant metastases stratified by sex and metastatic pathway is presented in Table 1.

**Table 1:** Clinical and histopathological characteristics.

	Pathway 1		Pathway 2		Pathway 3		*P* *
	Men%	Women %	Men %	Women %	Men %	Women %	
	n = 105	n = 151	n = 303	n = 246	n = 153	n = 120	
**Anatomical site**							<0.001 between sexes in pathways 1 and 2; 0.003 between sexes in pathway 3
Head and neck	15.2	15.2	17.5	12.2	17.8	23.3	
Trunk	42.9	13.2	52.1	25.6	64.1	42.5	
Upper extremities	10.5	9.3	10.6	16.7	7.8	12.5	
Lower extremities	31.4	62.3	19.8	45.5	10.5	21.7	
**Histological type**							NS between sexes
SSM	45.7	46.4	57.1	50.8	54.2	52.5	
NM	38.1	26.5	27.7	28.9	30.7	29.2	
LMM	5.7	9.9	5.3	5.7	4.6	7.5	
ALM	5.7	9.3	4.0	10.2	4.6	4.2	
Other	4.8	7.9	5.9	4.5	5.9	6.7	
**Tumor thickness in mm**							0.017 between sexes in pathway 1; NS between sexes in pathways 2 and 3
≤1.00	12.4	11.9	23.4	19.9	19.0	21.7	
1.01–2.00	29.5	35.8	29.7	29.3	32.0	35.8	
2.01–4.00	26.7	37.1	32.8	35.8	32.0	25.8	
>4.00	31.4	15.2	14.2	15.0	20.3	16.7	
Median (IQR)	2.6 (3.6)	2.1 (1.9)	2.0 (2.1)	2.1 (1.9)	2.2 (2.4)	1.7 (2.0)	†NS between sexes
**Ulceration**							NS between sexes
Yes	30.5	24.5	20.5	17.1	25.5	16.7	
No	69.5	75.5	79.5	82.9	74.5	83.3	
**Age**							NS between sexes in pathways 1 and 2; 0.002 between sexes in pathway 3
≤43 years	21.0	14.6	27.4	28.5	19.0	35.0	
44–60 years	28.7	29.8	40.6	36.2	45.1	27.5	
>60 years	52.4	55.6	32.0	35.4	35.9	37.5	

1,078 patients (561 male and 517 female) diagnosed from 1976 to 2008 as invasive primary cutaneous melanoma who developed metastases during follow up stratified by sex and metastatic pathway.

The funders had no role in study design, data collection and analysis, decision to publish, or preparation of the manuscript. No additional external funding was received for this study.

ALM, acral lentiginous melanoma; IQR, interquartile range; LMM, lentigo maligna melanoma; NM, nodular melanoma; NS, not significant; SSM, superficially spreading melanoma.

Pathway 1, primary tumor, then satellite/in-transit metastases; Pathway 2, primary tumor, then regional lymph node metastasis; Pathway 3, primary tumor, then distant metastasis.

* Pearson's χ^2^ test.

† Mann-Whitney test.

**Figure 1:**
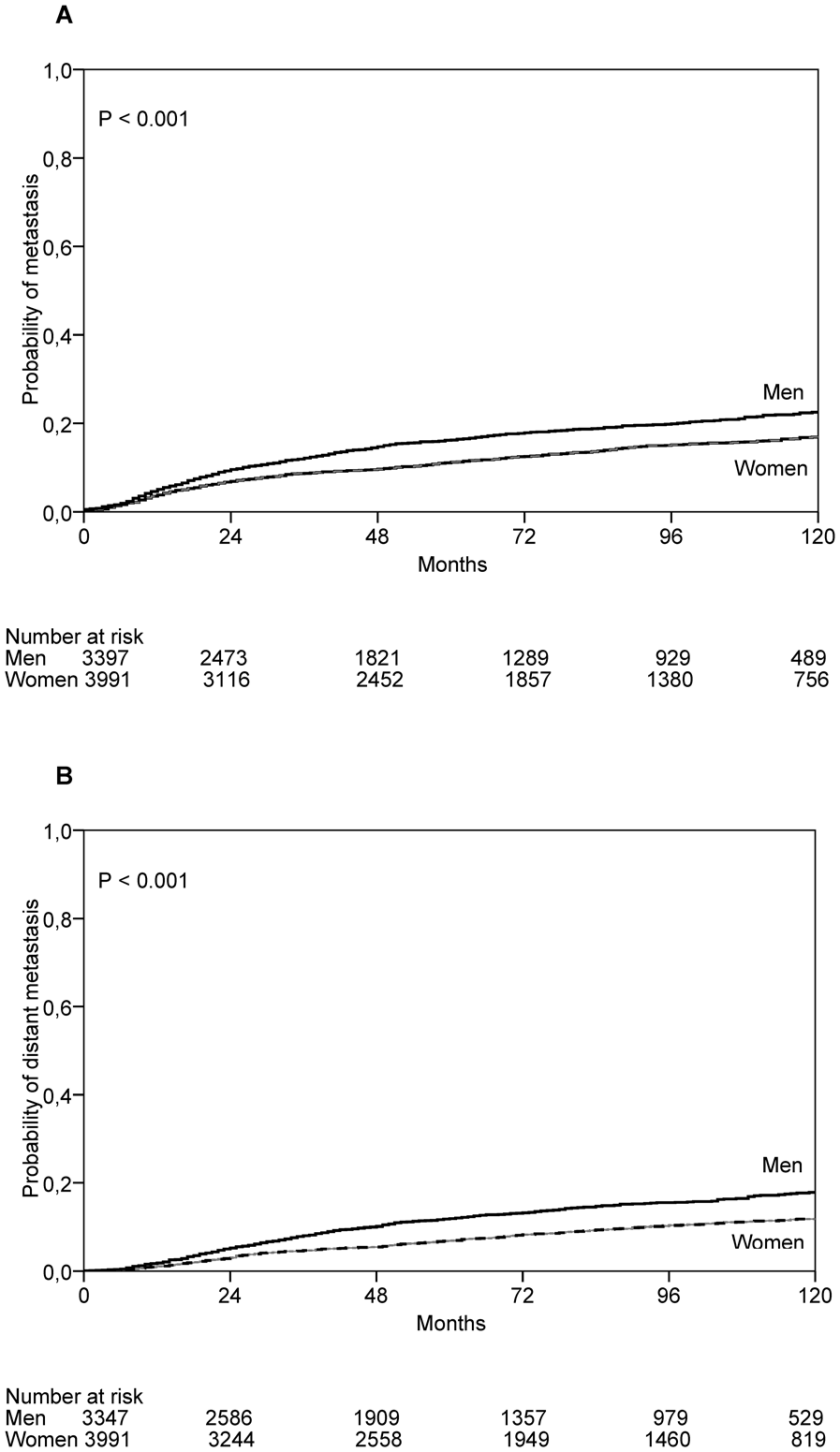
Probability for developing metastases. Probability for developing metastases (**A**), and distant metastases (**B**) after the diagnosis of primary cutaneous melanoma in 7,338 patients, 3,347 men and 3,991 women. *P* values are based on the log-rank test.

Using the Kaplan Meier method, probabilities for developing metastasis over a period of ten years was calculated, taking censored observations into account. The 10-year probability for developing metastases after the diagnosis of primary tumor was 22.8% (95% CI: 20.9–24.7) for men compared with 16.9% (95% CI: 15.4–18.4; *P*<0.001) for women (Figure 1A). The 10-year probability for developing distant metastases after the diagnosis of primary tumor was 17.8% (95% CI: 16.0–19.6) for men compared with 11.7% (95% CI: 10.4–13.1; *P*<0.001) for women (Figure 1B).

**Figure 2:**
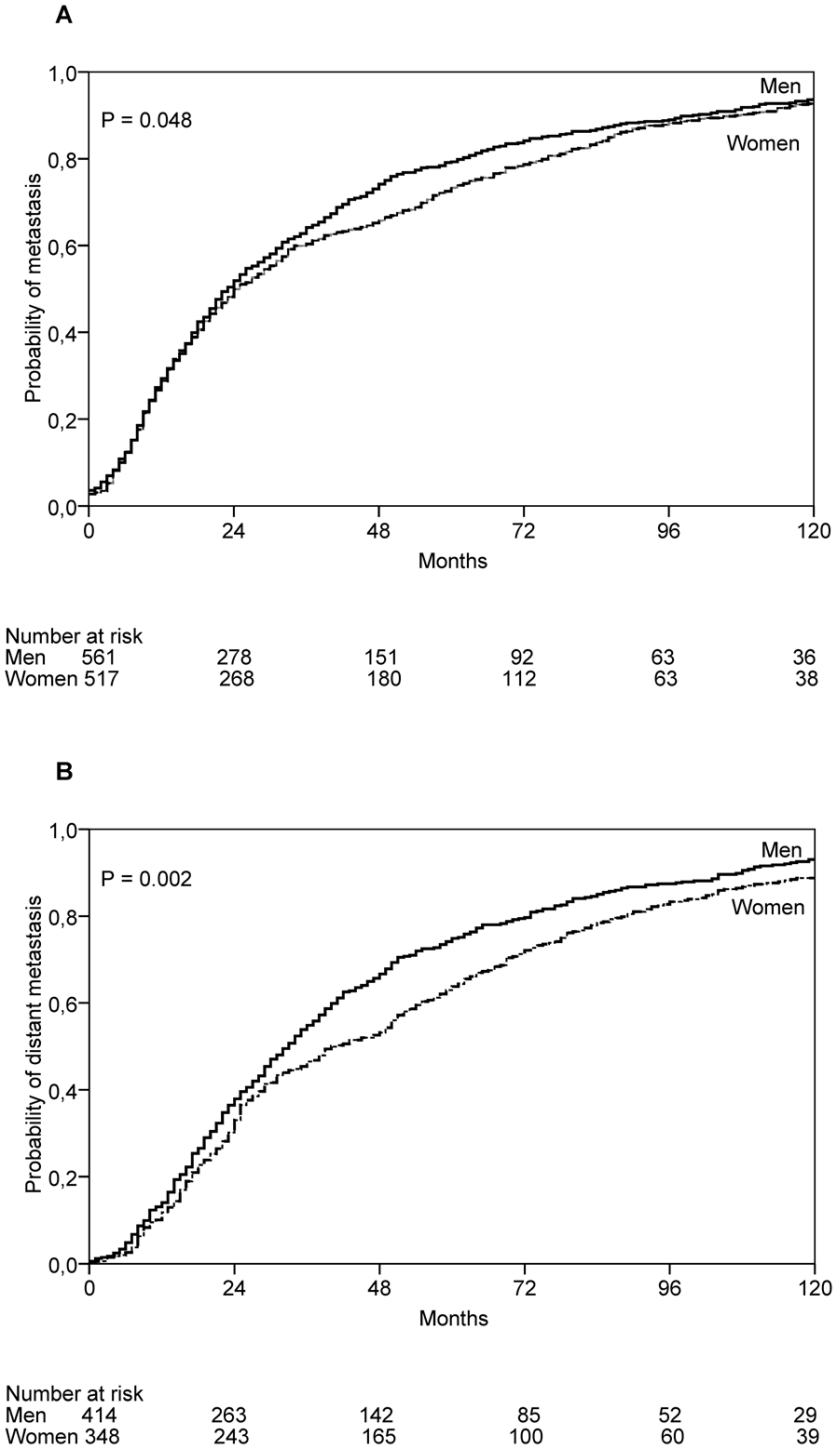
Probability for developing metastases. Probability for developing metastases (**A**), and distant metastases (**B**) in 1,078 patients (561 men and 517 women) who were diagnosed at the stage of primary cutaneous melanoma and then progressed by the time of analysis. *P* values are based on the log-rank test.

Women developed their first metastasis significantly later compared with men (*P* = 0.048). Median time to first metastasis was 25 months (IQR, 53) in women and 23 months (IQR, 38.5) in men (Figure 2A). Similarly, women developed distant metastasis significantly later compared with men (*P* = 0.002). Median time to distant metastasis was 40.5 months (IQR, 58.75) in women and 33 months (IQR, 44.25) in men (Figure 2B).

### Metastatic pathways

**Figure 3:**
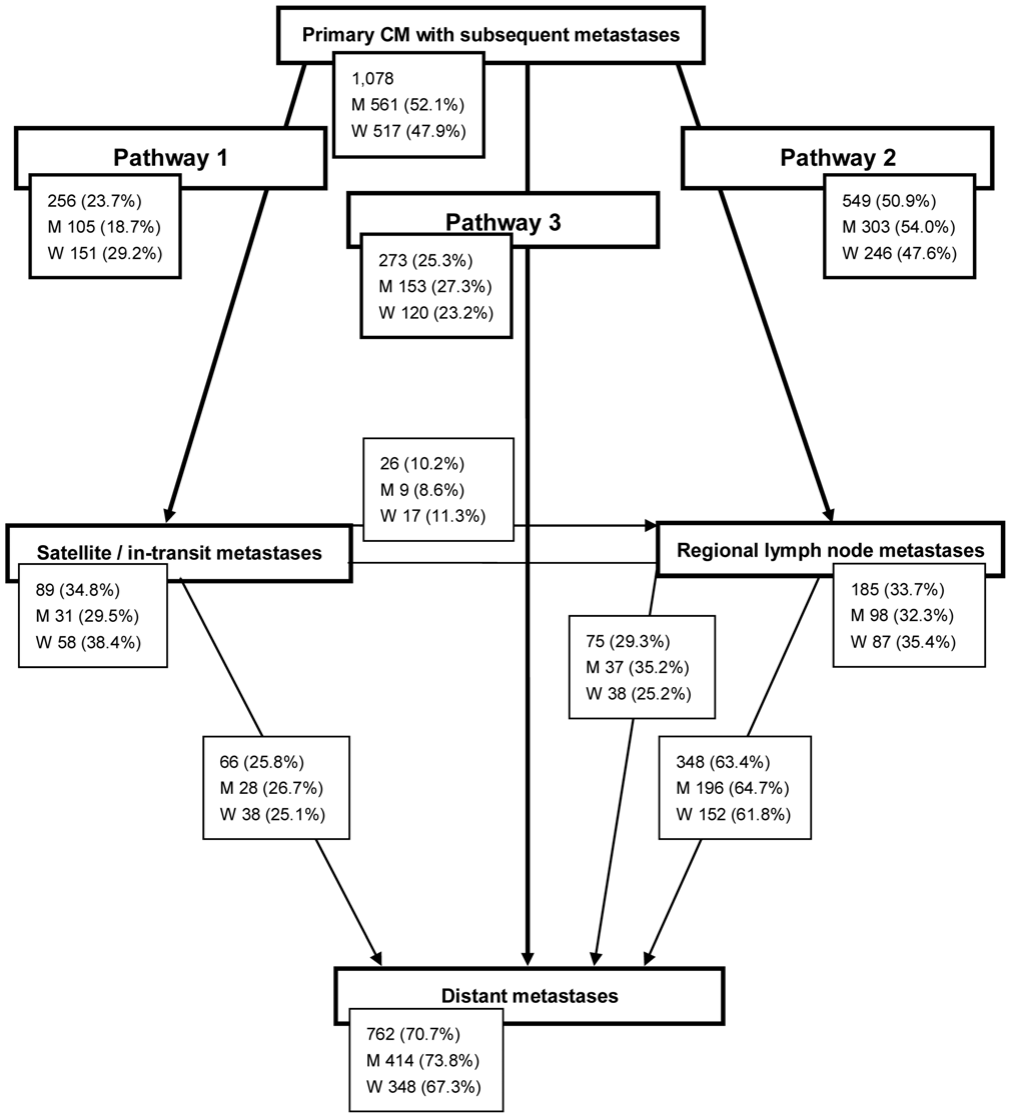
Three metastatic pathways of melanoma. Pathway 1 – primary tumor, then satellite/in-transit metastases, pathway 2 – primary tumor, then regional lymph node metastasis, and pathway 3 – primary tumor, then distant metastasis. M, men; W, women.

The most frequent site for the first metastasis was the regional lymph nodes (50.9%), followed by distant sites (25.3%). Satellite or/and in-transit metastases were the least frequent (23.7%). The metastatic pathways of the progression of CM were analyzed according to sex. A metastatic pathway was taken to be the first pathway of spread in disease progression. Three pathways were defined and are summarized in Figure 3: pathway 1 (primary tumor, then satellite/in-transit metastases), pathway 2 (primary tumor, then regional lymph node metastasis), and pathway 3 (primary tumor, then distant metastasis).

Development of satellite and in-transit metastasis was analyzed in detail. In 105 (18.7%) men and 151 (29.2%, *P*<0.001) women this was the first metastasis following diagnosis of primary tumor (Pathway 1). Of these patients 9 (8.6%) men and 17 (11.3%) women later on developed regional lymph node metastases, and 37 (35.2%) men and 38 (25.2%) women progressed first to regional lymph node and then to distant metastases. In 28 (26.7%) men and 38 (25.1%) women, distant metastases occurred directly after satellite or/and in-transit metastases. Men developed distant metastasis following pathway 1 after a median time of 29 months (IQR 39) compared to 52 months (IQR 67) in women (*P* = 0.002).

Direct regional lymph node metastasis developed in the majority of patients. This was observed in the majority of men (303, 54.0%) and women (246, 47.6%, *P* = 0.035; Pathway 2). Further progression of the tumor from this stage to the stage of distant metastasis was found in 196 (64.7%) men and 152 (61.8%) women. Men developed distant metastasis following pathway 2 after a median time of 30 months (IQR 43) compared to 32 months (IQR 51) in women (*P* = 0.1).

Direct distant metastasis from the stage of the primary tumor was observed in 153 (27.3%) men and 120 (23.2%, *P* = 0.13) women (Pathway 3). Men developed direct distant metastasis after a median time of 38 months (IQR 44) and women after a median time of 49 months (IQR 57, *P* = 0.4).

The anatomical site of tumor was the most important factor in the pattern of progression of the disease and there were significant differences between sexes (*P*<0.001 in pathways 1 and 2, and *P* = 0.003 in pathway 3). The primary melanoma was located on the lower-extremity in almost two thirds of women and one third of men who first progressed to satellite or in-transit metastases and only in 22% of women and 11% of men who first progressed to distant disease. Patients who progressed directly to distant sites had most frequently their primary tumors located on the trunk (64% of men and 43% of women). The second most frequent location of the primary melanoma in the case of direct distant metastases was head and neck. We did not observe any significant differences between sexes in the distribution of metastatic pathways for the different histological types, tumor thicknesses and presence of ulceration.

### Survival analysis

Patients with primary CM, who subsequently developed satellite/in-transit, regional lymph node or distant metastases as the first site of recurrence, were included into the survival analysis and were stratified by sex.

**Figure 4:**
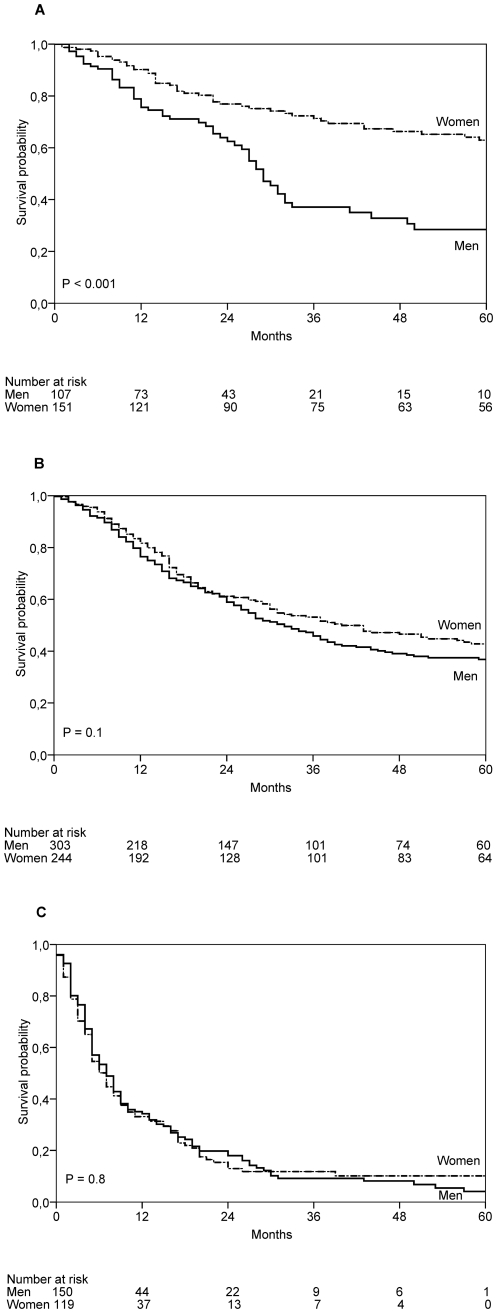
Survival after recurrence. Survival after recurrence in patients who progressed first to satellite/in-transit (**A**), regional lymph node (**B**), and distant metastases (**C**). *P* values are based on the log-rank test.

In 105 males and 151 females first metastasis occurred in local anatomical sites as satellite or/and in-transit metastasis. In these patients, five-year SAR probability differed significantly between sexes. It was 28.5% (95% CI: 17.0–39.9) for men and 63.0% (95% CI: 54.1–71.8; *P*<0.001) for women (Figure 4A). Median survival time of these patients was 19 months (IQR 22) for men and 33 months (IQR 69.75) for women.

In 302 male and 246 female patients their first metastasis developed in the regional lymph nodes. Five-year SAR probability in these patients was 36.8% (95% CI: 30.6–43.1) for men and 42.8% (95% CI: 35.9–49.7, *P* = 0.1) for women, and the difference was not statistically significant (Figure 4B). Median survival time of these patients was 23 months (IQR 37) for men and 27.5 months (IQR 53) for women.

In 153 men and 120 women their first metastasis developed at distant sites. Five-year SAR probability in these patients was 4.1% (95% CI: 0.1–8.2) for men and 10.1% (95% CI: 3.8–16.4, *P* = 0.8) for women, and this difference was likewise not statistically significant (Figure 4C). Median survival time of these patients was 6 months (IQR 11) for men, the same as for women (IQR 13).

During follow up, altogether 414 male and 348 female patients with primary CM at diagnosis eventually developed distant metastases in the course of the disease following all three metastatic pathways. SADR time was significantly longer in women compared with men. Male patients had 5.2% (95% CI: 1.4–2.5) five-year SADR probability compared with 15.3% (95% CI: 11.1–19.5; *P* = 0.008) for female patients. Median SADR-time was 7 months (IQR 11) for men and 8 months (IQR 14) for women. A total of 581 patients (322 men and 259 women) died of melanoma during the 10 years of follow-up time.

In the total cohort of 1078 patients who had developed metastasis, independent predictive factors for survival were analyzed. The results of the multivariate Cox regression analysis regarding SAR are presented in table 2. In the model showing the main effects of the variables, site of first metastasis was the most important prognostic factor (HR: 1.3; 95% CI: 1.0–1.6 for metastasis to the regional lymph nodes vs. satellite/in-transit recurrence and HR: 5.5; 95% CI: 4.2–7.1 for distant metastasis vs. satellite/in-transit recurrence, *P*<0.001). Other independent prognostic factors were sex (HR: 1.2; 95% CI: 1.0–1.5; *P* = 0.018), time to relapse after primary diagnosis (HR: 0.996; 95% CI: 0.994–0.998; *P*<0.001), and decade of diagnosis (HR: 0.8; 95% CI: 0.7–0.9; *P*<0.001). Age, anatomical site of primary melanoma, tumor thickness, Clark's level of invasion, histological ulceration, and histological subtype were not important factors in the equation, and seemingly do not play any role once metastasis has developed.

**Table 2:** Multivariate predictors of survival after first recurrence.

	HR (95% CI)^a^	*P* b
**Sex** (male vs. female)	1.2 (1.0–1.5)	0.018
**Age** (years)	1.002 (0.996–1.008)	0.5
**Anatomical site**		
Head/neck	1.0	0.2
Trunk	1.3 (1.0–1.6)	0.09
Upper extremities	1.1 (0.8–1.5)	0.7
Lower extremities	1.0 (0.8–1.4)	0.9
**Tumor thickness** (mm)	1.0 (0,96–1.04)	0.9
**Clark** (II/III vs. IV/V)	0.9 (0.7–1.0)	0.1
**Ulceration** (yes vs. no)	1.1 (0.9–1,4)	0.3
**Histological type**		
SSM	1.0	0.5
NM	1.0 (0.8–1.3)	0.8
LMM	1.2 (0.8–1.8)	0.3
ALM	1.1 (0.7–1.6)	0.7
Other	0.8 (0.5–1.2)	0.2
**Site of first metastasis**		
Satellite/in-transit	1.0	<0.001
Regional lymph node	1.3 (1.0–1.6)	0.07
Distant	5.5 (4.2–7.1)	<0.001
**Recurrence free survival time** (months)	0.996 (0.994–0.998)	<0.001
**Decade of diagnosis**	0.8 (0.7–0.9)	<0.001

ALM, acral lentiginous melanoma; CI, confidence interval; HR, hazards ratio; LMM, lentigo maligna melanoma; NM, nodular melanoma; SSM, superficially spreading melanoma.

a HR with its 95% CI.

b Wald test significance; *P* values listed at the level of the reference categories (HR = 1.0) represent significance for the variable as a whole, all other *P* values represent significance of the category specified as compared to the reference category.

## Discussion

It is well established, that gender is an independent prognostic factor in primary invasive melanoma and that female patients have a more favorable outcome compared to male patients when other prognostic features of the primary tumor are comparable. This study addresses for the first time the differences in the natural course of cutaneous melanoma between men and women. The results of this study show that women metastasize less often and develop all kinds of metastases later as compared with men. Furthermore, women show a different pattern of metastasis with a strikingly higher percentage of the prognostically favorable satellite/in-transit metastasis. Finally, women have a significantly higher five-year survival probability once distant metastasis has developed.

The probability for developing metastasis and distant metastasis after the diagnosis of primary tumor in our series of patients was significantly higher for men compared with women. Additionally, if disease progression had taken place, women developed their first metastasis as well as distant metastasis significantly later compared with men.

It is well known that approximately two thirds of patients who develop clinical metastases following a primary CM initially present with locoregional metastases and one third initially present with distant metastases [10]. In our study group the most frequent site for the first metastasis was the regional lymph nodes (50.9%), followed by distant sites (25.3%) and satellite or/and in-transit metastases (23.7%). In significantly more women compared with men the first metastasis following diagnosis of primary tumor took the form of satellite or/and in-transit metastases. The majority of patients exhibited direct regional lymph node metastasis with significant predominance of men. We observed no difference between sexes in frequency of direct distant metastasis from the stage of the primary tumor. About one third of patients, who first developed satellite/in-transit or regional lymph node metastasis, remained at the stage of locoregional metastasis during follow up, while approximately two thirds of patients (more men than women) exhibited further progression to distant sites. Therefore, the pattern of metastasis differed between sexes. There was a clear tendency for women to recur locally and they experienced further progression to distant sites less often then men. Furthermore, time from primary tumor diagnosis to distant metastasis after satellite/in-transit metastases (Pathway 1) was significantly longer in female compared with male patients. There were no sex related differences in time appearance of distant metastasis in other two metastatic pathways, namely after lymph node metastasis as the first site of recurrence or direct distant metastasis after the primary tumor.

Interestingly, in several studies patients were reported to reach the stage of distant metastasis after a similar period of time (median approximately 25 months) irrespective of the metastatic pathway [10], [14], [15]. At present, there are several models that attempt to explain how melanoma spreads. The stepwise spread model, in which the melanoma spreads first by a lymphatic pathway towards the regional lymph nodes, from where systemic dissemination is initiated with a certain time lag does not explain the fact that about a third of patients progress directly to distant sites [16], [17]. A model of simultaneous spread assumes that the primary tumor would metastasize simultaneously by lymphatic and hematogenous pathways, and lymph node involvement would therefore be a marker of systemic disease. This model does not fit with the fact that regional lymph node dissection is curative in about one third of cases [17], [18]. The third model of differential spread is based on the assumption that some melanomas will not have the biological potential to metastasize, others will only be able to metastasize to regional lymph nodes or to metastasize to both regional lymph nodes and distant organs, or some melanomas will only be able to metastasize at the systemic level. Tumor progression in CM is a complex and multistep process including proliferation, neovascularization, lymphangiogenesis, invasion, circulation, embolism, and extravasation. This last model can explain some premises about the natural history of melanoma that cannot be explained by the other two models, namely that some melanoma patients can achieve complete and long-lasting remission following therapeutic lymph node resection, but not all of them because lymph node and distant metastases can develop at the same time. Furthermore, this model can explain why a negative finding in the sentinel lymph node biopsy does not provide an absolute guarantee of survival [17], [19]. In our study group of patients women more frequently metastasized locally as satellite/in-transit metastasis and further progression to distant site was noted after a significant time lag as compared with men.

Analyzing the distribution of various clinical and histopathological characteristics of melanoma at the time of the first diagnosis in patients who progressed to the stage of locoregional or distant metastases, the anatomical site of tumor was the most important factor influencing the pattern of progression of the disease and there were significant differences between sexes. The primary melanoma was located on the lower-extremity in almost two thirds of women and one third of men who firstly progressed to satellite or/and in-transit metastases. Patients who progressed directly to distant sites most frequently had their primary tumors located on the trunk (64% of men and 43% of women). The second most frequent location of the primary melanoma in the case of direct distant metastases was head and neck. Sex-related differences in metastatic pathways could be related to sex-related differences in the site of the primary tumor. It is well known that melanomas on the lower extremities, which are more common in female, have a better prognosis than tumors located on the trunk, which are more commonly seen in male patients [9], [10].

The prognostically unfavorable locations of primary tumors were proposed for classification as TANS (trunk, upper arm, neck, scalp) or BANS (back, upper arm, neck, scalp) regions [20], [21]. It has been speculated that lymphatic drainage may be responsible for differences in the clinical course of melanomas at different anatomical sites. The length of the lymph tracts and the number of lymph nodes to be passed before the lymphatic fluid reaches the blood circulation at the venous angle in the upper thorax could be decisive factors for the defense of the immune system of the host against metastasizing tumor cells. Therefore, better prognosis for the lower extremity tumors might be due to the higher barrier to metastatic cells presented by the longer lymphatic vessels and greater number of lymph nodes before reaching systemic circulation [9], [10].

Site of first metastasis was the most important prognostic factor of survival after recurrence in multivariate analysis in our series of patients, followed by sex, time to diagnosing the first metastasis and decade of diagnosis of the primary tumor. Hazard rate for dying of melanoma was 1.3 if the patient exhibited metastasis to the regional lymph nodes vs. satellite/in-transit recurrence, and 5.5 for distant metastasis vs. satellite/in-transit recurrence. Satellite or in-transit metastases after the primary tumor which are prognostically favorable were significantly more common in women. Furthermore, female patients had significantly longer time to diagnosing the first metastasis, also explaining their better survival. The finding that site of first metastasis is the most important prognostic factor of survival after first recurrence has been reported previously [22].

Five-year SAR probability for patients from our collective in whom first metastasis occurred in local anatomical sites as satellite or/and in-transit metastasis differed significantly between sexes. However, it did not reach statistical significance in patients who developed their first metastasis in the regional lymph nodes, neither in patients who developed their first metastasis at distant sites. Despite the finding that there were no differences between sexes in survival after first recurrence in the regional lymph nodes or at distant sites, SADR probability in patients who eventually developed distant metastases in the course of the disease following all three metastatic pathways was significantly higher in women compared with men.

Favorable natural course of melanoma in women with a lower chance of melanoma progression after the diagnosis of primary tumor and later development of metastases in the case of disease progression is crucial for better survival of female patients. Additionally, different pattern of metastasis with more frequent local recurrencies in the form of satellite/in-transit metastasis explains their better survival. Even women who eventually progress to the stage of distant disease have better five-year survival compared with men.

The molecular basis for sex differences in the natural course of melanoma remains undefined. Sex steroids have been suggested as playing a role in melanoma progression, but the majority of evidence now is that melanoma is not hormone dependent [23]–[25]. There may be sex differences in immunity, which could also explain the observed differences in melanoma progression. Sexual dimorphism in the immune system of female and male patients suggested by sex differences in the incidence of autoimmune diseases could be of relevance to natural course and surviving melanoma [26], [27]. Another proposed explanation of the female advantage could be sex differences in oxidative stress caused by radical oxygen species, which are known to be able to promote metastasis through a wide variety of mechanisms. Worse outcome of male melanoma patients may be explained by the lower amounts of antioxidant enzymes expressed in men, resulting in more oxidative stress than in women [28].

Present study has several limitations. It was not planned in a statistical manner, but it is the evaluation of cancer registry data. Also the issue of multiple testing has to be acknowledged. On account of these limitations, an independent verification of this study results is required. Unfortunately, CMMR had no data regarding gynecological details and the mitotic rate of primary tumor, which was recently shown to be an independent prognostic variable in CM patients. The presence of ulceration may have been underreported during the early years of the study period. The sentinel lymph node status of our patients diagnosed during the early years of the study was not available. Patients with clinically undetectable lymph node micrometastasis were classified into stages I and II before 1996 and afterwards into stage III melanoma, and were therefore excluded from the present analysis. In addition, we had no details regarding the number of involved lymph nodes in patients that developed regional lymph node metastasis, about systemic treatments administered to our patients, and about socioeconomic factors that are known to influence the outcome. On the other hand, we had high-quality data with negligible rates of unknown or missing events and almost complete long-term follow-up data for our entire sample. Although our sample is not population based, it is largely representative of the situation in southern Germany.

In conclusion, men had higher probability for developing metastases and distant metastases after the diagnosis of primary tumor. Additionally, if disease progression took place, women developed their first metastasis as well as distant metastasis significantly later compared with men. When the metastatic pathways are taken into consideration, women more frequently progressed first to local sites in the form of satellite or in-transit metastases and men more frequently exhibited direct regional lymph node metastasis. About two thirds of these patients, more men than women, showed further progression to distant sites. There were no differences between sexes in the occurrence of direct distant metastasis after the primary invasive CM. Site of first metastasis was shown to be the most important prognostic factor of survival after recurrence in multivariate analysis. This analysis shows, that different factors like delay in the development of metastasis and different pathways of metastasis contribute to the more favorable survival of women, even when metastasis has already developed. These findings may stimulate further research concerning the potential role of molecular factors responsible for this sex differences and differences in survival of patients with cutaneous melanoma in general.
